# Restored Vision

**Published:** 1844-07

**Authors:** 


*Restored Vision.*—A remarkable case of perfect restoration of vision, after a total blindness of many months, has been brought to our notice within a few days, which reflects the highest credit on the skill of Dr. Davenport, of this city. The patient had been dreadfully injured by an explosion of powder, which wholly destroyed one eye, and so deranged the other that no encouragement had been given, before he fell under the cognizance of this gentleman, that light would again shine in upon the retina. Owing to extensive inflammation, the pupil was obliterated; and no one less ardent or devoted to the interests of his profession, would have indulged a hope of opening a new window through the thickened semi-transparent tunics of such an eye. A new pupil was formed—and, lo! a cataract was found. Dr. D. couched the unfortunate man—and, to their mutual delight, vision was restored. A beautiful colored drawing has been executed, illustrative of the external appearances before the operation, which should be published for a guide in similar cases. We are expecting the details at a convenient time.

*Boston Med. and Surg. Jour.* for May.

